# Piezoelectric and Dielectric Response of BaTiO_3_/PVDF-TrFE Composites with High β‑Phase Content

**DOI:** 10.1021/acsapm.5c00620

**Published:** 2025-06-06

**Authors:** Andrea Otero, María Jesús Sayagués, Francisco Javier Romero, Francisco José Gotor, Rocío Moriche

**Affiliations:** † Departamento de Física de la Materia Condensada, Facultad de Física, 16778 Universidad de Sevilla-ICMS, Avda. Reina Mercedes, s/n, Sevilla 41012, Spain; ‡ Instituto de Ciencia de Materiales de Sevilla (ICMS), CSIC-US, Américo Vespucio, 49, Sevilla 41092, Spain

**Keywords:** PVDF-TrFE, β-phase, barium titanate, piezoelectric properties, dielectric properties

## Abstract

The search for flexible piezoelectric materials to build adaptable sensors, electronics, and nanogenerators has become a key area of interest. The addition of piezoceramic particles to piezoelectric polymers, such as the copolymer poly­(vinylidene fluoride-trifluoroethylene) (PVDF-TrFE), is one of the strategies used to enhance the piezoelectric response. In this work, the effect of BaTiO_3_ content on the β-phase formation, crystallization, and piezoelectric and dielectric properties of the polymer-based composites is investigated. High-energy ball milling was used as an effective, greener technique to achieve well-dispersed mixtures compared to those obtained using organic solvents. During the dispersion process, amorphization and reduction of the crystalline domain size occur. After compression molding and postprocessing, the crystallinity was recovered and was strongly dependent on the filler content. Although significant differences in the β-phase fraction were not observed, conformational defects are induced with high BaTiO_3_ contents. The interlayer distances became smaller due to the presence of the ceramic particles after compression molding and remained almost unchanged after postprocessing. For the composites, the minimum voltage required to obtain a measurable piezoelectric coefficient (*d*_*33*_) was significantly reduced compared to neat PVDF-TrFE, even for low contents, which is key for real applications. Three different piezoelectric behaviors were found depending on the BaTiO_3_ fraction. For composites with 40 vol %, where both matrix and filler contribute to the overall piezoelectric response, the use of a two-step poling method induced a synergistic effect with an increase in *d*_*33*_ of ∼180%. However, the relaxation of the ceramic contribution after 24 h returns the value of *d*_*33*_ to that obtained by applying a one-step poling strategy.

## Graphical abstract

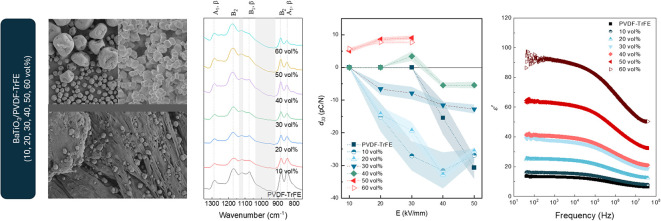

## 1 Introduction

During the past decade, the interest in finding flexible piezoelectric materials to build adaptive pressure sensors, electronics, and nanogenerators for use in wearable electronics and self-powered devices, among others, has become a key field.^1,2^ In this context, poly­(vinylidene fluoride) (PVDF) has attracted the interest of the research community^3–5^ not only because of its flexibility, chemical stability, and easy processing but also because, through different strategies of synthesis, processing, and chemical modification (formation of copolymers, terpolymers, etc.), it is possible to achieve different behaviors in terms of ferroelectric, dielectric, and piezoelectric properties.^6^ This polymer can crystallize in different conformations (α, β, γ, δ, and ε) as a result of internal rotation and steric hindrance.^7^ The nonpolar α-phase with trans–gauche conformation (TGTG) is the most stable in PVDF, so great efforts are made to stabilize the polar β-phase with all-trans conformation (TTTT), which shows the stronger piezoelectric response.^3^ Among the strategies to maximize the presence of the polar phase, structural modifications have been considered to induce the conversion of α- to β-phase, achieving a 1.5-fold improvement in the piezoelectric response.^8^ In this context, the incorporation of trifluoroethylene (TrFE) units into PVDF helps to stabilize the β-phase and, consequently, the copolymer PVDF-TrFE has been positioned as more promising.^9^

Other approaches to promote β-phase crystallization and increase crystallinity, which also have a significant impact on piezoelectric behavior,^3^ include processing-related strategies, such as mechanical stretching of the polymer and the application of high electric fields during conformation.^10^ For example, the high electric field used in electrospinning to produce PVDF nanofibers forces the molecular dipoles to be oriented, resulting in high fractions of β-phase.^11^

Another route is the addition of different fillers (electrically conductive and/or piezoceramic particles).^11–14^ Regarding this last strategy, an appropriate composite design is required to enhance the piezoelectric performance of this type of materials.^15^ Singh et al.^13^ obtained an output voltage and short-circuit current approximately 2.5 and 3.5 times higher, respectively, using NaNbO_3_ and reduced graphene oxide (rGO) as fillers because they favor the alignment of the β-phase dipoles. Sahoo et al.^15^ reported an improvement in the dielectric and ferroelectric behavior of PVDF-TrFE by incorporating Ca-doped ZnO. Lin et al.^16^ found that the presence of BaTiO_3_ particles in PVDF delays the relaxation of the molecular chains and favors the TTTT conformation. Kubin et al.^17^ reported that although the incorporation of BaTiO_3_ from 5 to 20 wt % does not directly affect the piezoelectric performance of the mats, it does increase the crystallinity due to nucleation caused by the filler, which impacts the response.

In most of these works, the contribution of the piezoelectric response of the filler is not addressed, probably due to the low filler fraction (<10 vol % in the case of BaTiO_3_), and often the poling in these composite materials is carried out in a single step under high electric fields ranging from 10 to 100 kV/mm,^18,19^ which means that the piezoelectric response of the piezoceramic filler and the PVDF or PVDF-TrFE matrix may be opposite. Only a few authors^20^ have pointed out the two-step poling to achieve the synergy of both the filler and the matrix. Therefore, further studies are needed to analyze the influence at higher concentrations and to clarify the conditions at which the intrinsic response of piezoceramic fillers becomes significant for the overall piezoelectric response.

The aim of this work is to analyze the influence of a piezoelectric nanoceramic incorporation (tetragonal BaTiO_3_) into a PVDF-TrFE matrix on the final structure and piezoelectric properties of the formed composite. Unlike other works, it includes a wide range of filler fractions (from 10 to 60 vol %) to elucidate how the intrinsic piezoelectric properties of the filler can condition the piezoelectric performance of the composite material.

## 2 Materials and Methods

PVDF-TrFE with a TrFE content of 25 mol % was used as the polymeric matrix. Powder-form PVDF-TrFE *Piezotech FC25* was purchased from *Arkema*. Tetragonal BaTiO_3_ from *Nanografi Nano Technology,* with an average particle size of <500 nm, was used as a filler in contents of 10, 20, 30, 40, 50, and 60 vol %. Densities used for mass calculations were 1.89 and 6.02 g/cm^3^, respectively.

High-energy planetary ball milling was used as the dispersion method (*Pulverisette 7*, *Fritsch*). The dispersion process was carried out under dry conditions to avoid the use of solvents. A steel jar with a 45 mL volume (15 mm diameter), together with 5 tungsten carbide balls of 15 mm diameter (ball-to-powder ratio (BPR) equal to 27), was used to prepare 6 g of the composite powder. The process consisted of 20 cycles of 10 min of milling and a 10-min pause at a speed of 500 rpm to avoid excessive heating.

Compression molding was used to obtain disk-shaped samples in a uniaxial hydraulic press (*CIP-15TA, MTI Corp*) with coupled heating plates (*GS15515, Specac*). Molding was performed at a pressure of 15 MPa at 200 °C for 15 min, with heating and cooling rates of 5 °C/min, to produce samples with thicknesses of 1.00 ± 0.09 mm. The samples were then subjected to thermomechanical postprocessing. A pressure of 20 MPa was applied at 140 °C for 15 min, with heating and cooling rates of 5 °C/min. In both processes, the pressure was maintained during cooling until the temperature reached 60 °C. The macroscopic strain was calculated as the unitary reduction in thickness, which ranged from 0.15 to 0.40 mm, depending on the filler content.

Changes in the phases formed were monitored throughout the process by attenuated total reflection Fourier transform infrared spectroscopy (ATR-FTIR) using an *Invenio-X* from *Bruker*. *SpectraGryph 1.2* was used to evaluate the results. Absorbance spectra were recorded in the wavelength range of 400–1500 cm^–1^.

Differential scanning calorimetry (DSC) was used to characterize the crystallinity, as well as the Curie (*T*_c_) and melting (*T*_m_) temperatures, in an *SDT Q600II* from *TA Instruments*. The tests were performed from −20 to 150 °C at a heating rate of 10 °C/min in air. The degree of crystallinity (%*X*_*c*_) was calculated according to the equation:^9,17,21^

1 $$\%X_{c}=100\times \left[\frac{\Delta H_{m}}{\Delta H_{m}^{{}^{\circ}}\times W_{\mathrm{PVDF-}\mathrm{TrFE}}}\right]$$

where Δ*H*_*m*_ is the melting enthalpy, and Δ*H*_*m*_° is the melting enthalpy considering a fully crystalline (100%) PVDF-TrFE (104.7 J/g, according to ref.^22^) and *W*_PVDF‑TrFE_ is the net weight fraction of the polymer in the composite material.

X-ray diffraction (XRD) was used for structural characterization in a *Philips X’Pert Pro.* The diffraction patterns were scanned from 18° to 21.5° (2θ) in step-scan mode with a step size of 0.03° and a counting time equivalent to 300 s/step. Calculations were performed using *X’Pert High Score* software. The crystalline domain size (*D*) was obtained by the Scherrer equation.

Morphological features and filler dispersion were analyzed by field emission gun scanning electron microscopy (SEM) on an *FEI TENEO* (*FEI Company*) operating at 2 kV. The samples were coated with a 6 nm platinum layer to avoid electrical charging.

Poling was carried out in a silicone oil bath under a DC electric field by using a high-voltage power supply (+30 kV/2 mA) provided by *PolyK*. Different poling voltages and durations were used depending on the material to analyze their effect on the piezoelectric response. The applied field varied from 4 to 80 kV/mm, and the duration ranged from 5 to 30 min. After removing the poling voltage, the top and bottom surfaces were short-circuited by using aluminum foil. The piezoelectric coefficient (*d*_*33*_) was then measured using a Berlincourt piezo *d*_*33*_ meter from *PolyK,* equipped with a static force sensor. The *d*_*33*_ values were measured immediately after poling (as-poled samples) and again after 24 h. The reported *d*_*33*_ value is an average of 20 measurements taken at different points on the surface: 10 along the poling direction and 10 along the opposite direction.

Dielectric properties were measured in unpoled samples using an Agilent 4294A in a frequency range of 50 Hz at 0.5 V.

## 3 Results and Discussion

### 3.1 Influence of BaTiO_3_ and Processing on the β-Phase Crystallization

Figure 1 shows representative SEM micrographs of the as-received PVDF-TrFE and BaTiO_3_ powders, as well as the as-milled composite powders (20, 40, and 60 vol %). The as-received PVDF-TrFE powder has a spheroidal geometry with a bimodal size distribution. The BaTiO_3_ powder consists of particles approximately 100–500 nm in size. After the dispersion process, the polymer morphology evolves into a planar shape due to the high stresses induced during high-energy planetary ball milling, with the size decreasing as the BaTiO_3_ content increases, as ceramic particles also act as a process-controlling agent (PCA). The high shear forces and the presence of the submicrometric hard filler during milling also cause fibrillation and cavities in the polymer matrix, features that disappear after molding. The BaTiO_3_ is homogeneously dispersed throughout the polymer matrix, and neat areas are not found. The presence of BaTiO_3_ agglomerates is also not observed. These results confirm that ball milling is a suitable and more environmentally friendly technique for obtaining well-dispersed mixtures for developing composite materials compared with methods using organic solvents.

**Figure 1:**
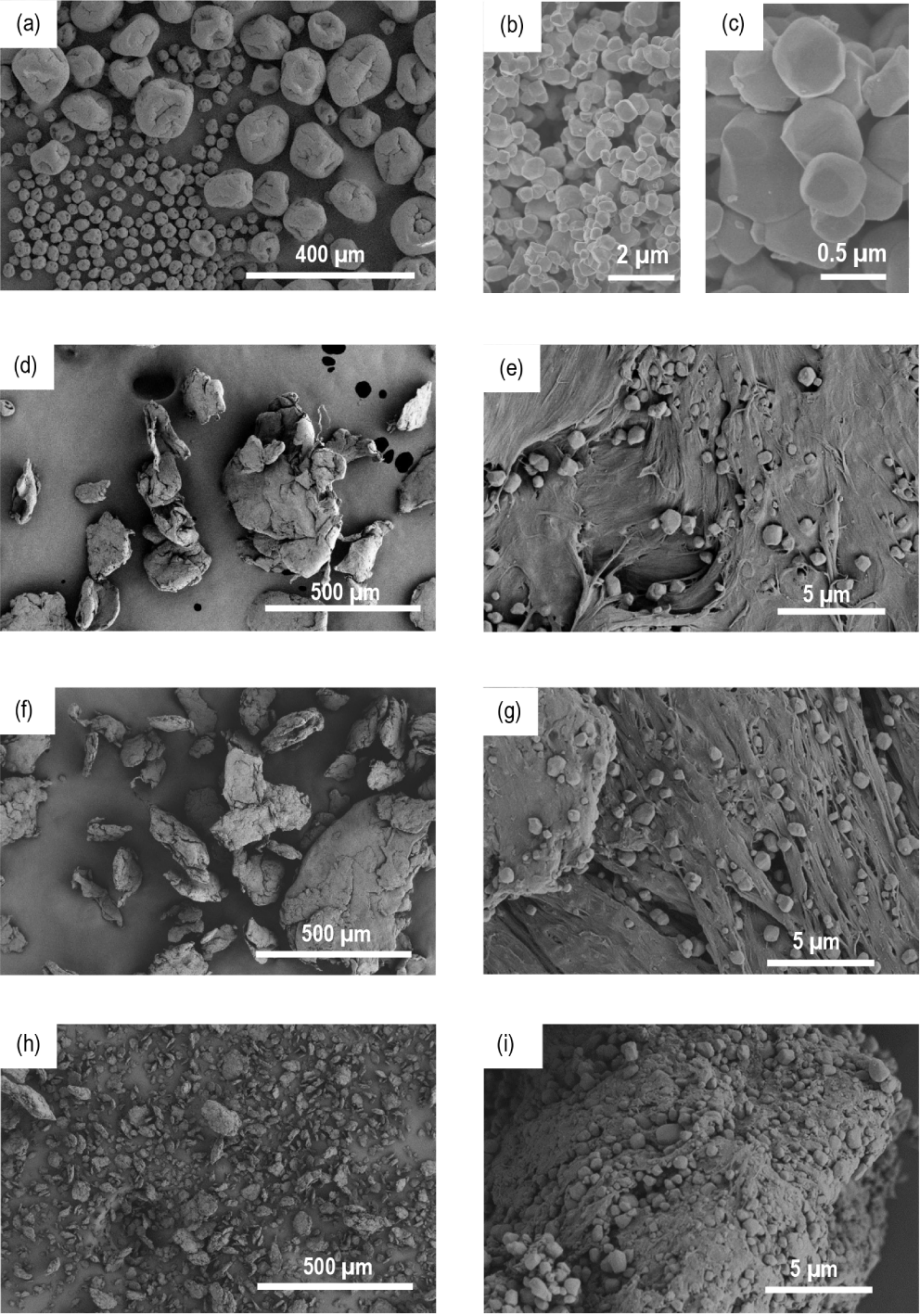
Morphology of the powder (a, b, c, d, f, h) and microstructure (e, g, (i) of (a) PVDF-TrFE, (b, c) BaTiO_3_, (d, e) 20, (f, g) 40, and (h, (i) 60 vol % BaTiO_3_-composites.

In addition to the morphological features, the dispersion process can also affect the structure of the PVDF-TrFE matrix. The evolution of the FTIR spectra after each processing step is shown in Figure 2, where the bands are labeled with the corresponding mode. As previously reported by other authors, in all the studied conditions, the formation of a crystalline phase similar to the electroactive β-phase (all-trans) of the PVDF can be detected by bands at 845, 1076, and 1289 cm^–1^.^23–25^ The bands observed at 883, 1183, and 1402 cm^–1^ are the main bands of the PVDF, which are barely sensitive to the crystal structure, and the one at 1124 cm^–1^ has been reported to appear due to specific vibrational modes of the TrFE units,^26^ although other authors have related it to a phase similar to the γ-phase of the PVDF or modes resulting from tt and tg segments of the PVDF.^27^ Resende et al.^27^ have stated that, although the β-phase is predominant in PVDF-TrFE copolymers, segments with tttg and tg conformations could be present in a defective all-trans polymer chain.

**Figure 2:**
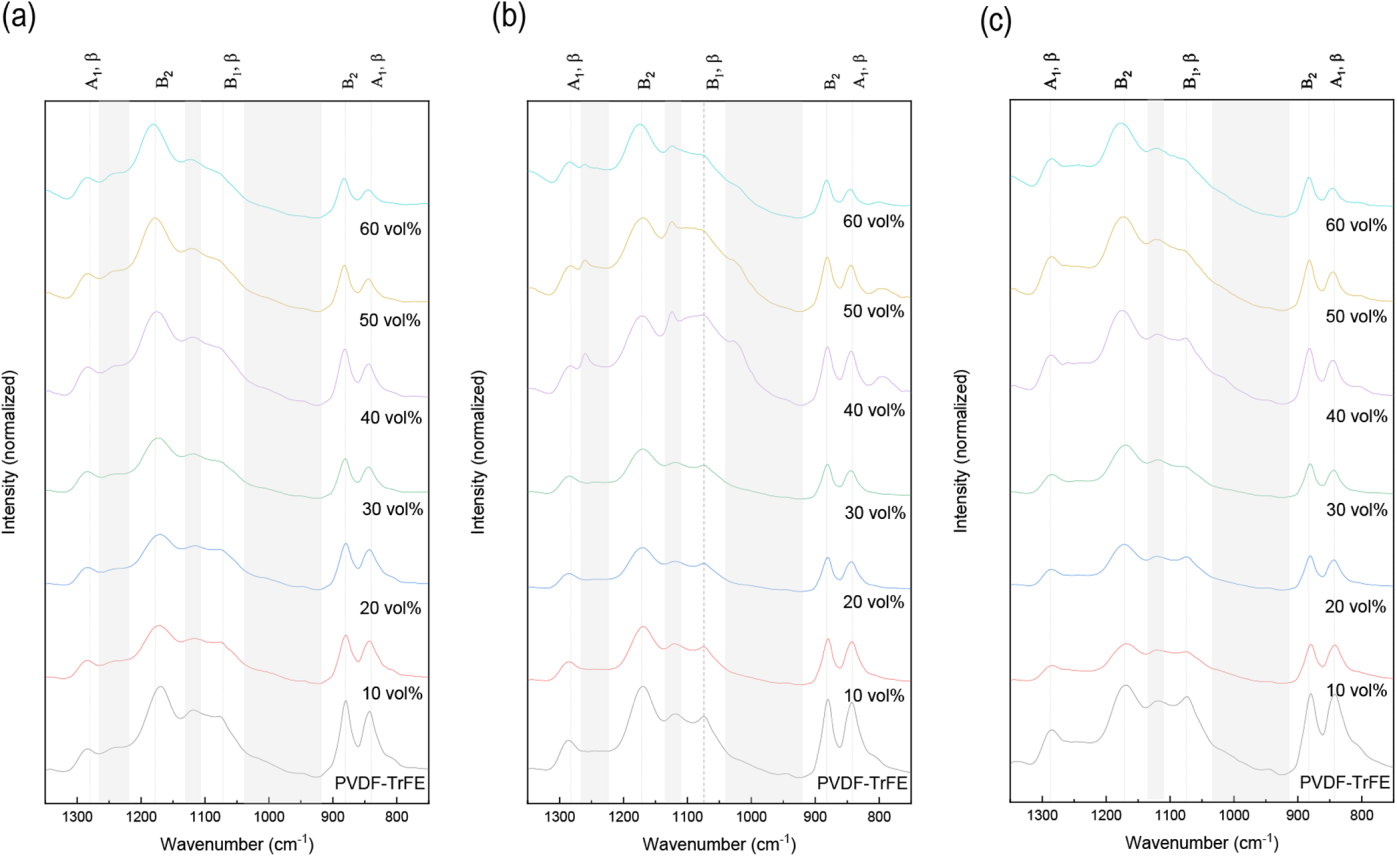
FTIR spectra of (a) powder form, (b) compression molded, and (c) postprocessed samples of PVDF-TrFE and composites (10, 20, 30, 40, 50, and 60 vol %).

In the powder form (Figure 2a), an additional band at 1234 cm^–1^ is present, which Arrigoni et al.^26^ have reported to be related to conformational disorder. With the addition of low BaTiO_3_ fractions and after the dispersion process, this band becomes negligible. Additionally, the specific bands associated with the β-phase are less significant at contents above 40 vol %, so the fraction of the ordered all-trans conformation diminishes. This fact is in contrast with the results obtained by Kubin et al.,^28^ who observed that the β-phase fraction is almost independent of the BaTiO_3_ content, ranging from 95.4 to 98.4%, which is attributed to a more significant impact of the incorporation of TrFE units in the stabilization of this phase, but they used contents below 20 wt % (<10 vol %).

After compression molding, which involves melting and recrystallization, the band at 1234 cm^–1^ disappears for all of the materials, indicating the reduction of conformational disorder. However, a new band at 1220–1250 cm^–1^ can be observed for composites reinforced with 40, 50, and 60 vol % (Figure 2b), which could be related to the contribution of disordered domains.^26,29^ After postprocessing, this band disappears due to the crystallinity recovering as a result of the reduction of these disordered domains. Additionally, the intensity of the band at 1289 cm^–1^ increases after postprocessing, as observed in Figure 2c, reinforcing the idea of increased crystallinity. Prabu et al.^29^ have likewise reported that an increase in crystallinity results in larger bands at 1291 and 1399 cm^–1^. Although the recovery of crystallinity is observed in all cases, the relative intensity of the specific bands associated with the β-phase is still less meaningful at contents above 40 vol %, and therefore, the fraction of the ordered all-trans conformation remains lower in highly filled composite materials.

These changes induced by the different processing steps were also analyzed by XRD (Figure 3). The calculated parameters can be found in Table S1. In all the XRD patterns where a peak is observed, it is located at 2θ ∼ 20°, which is associated with the (110) and (200) reflections of the β-phase (*d* ∼ 0.28 nm),^19,30^ and this is consistent with the FTIR results.

**Figure 3:**
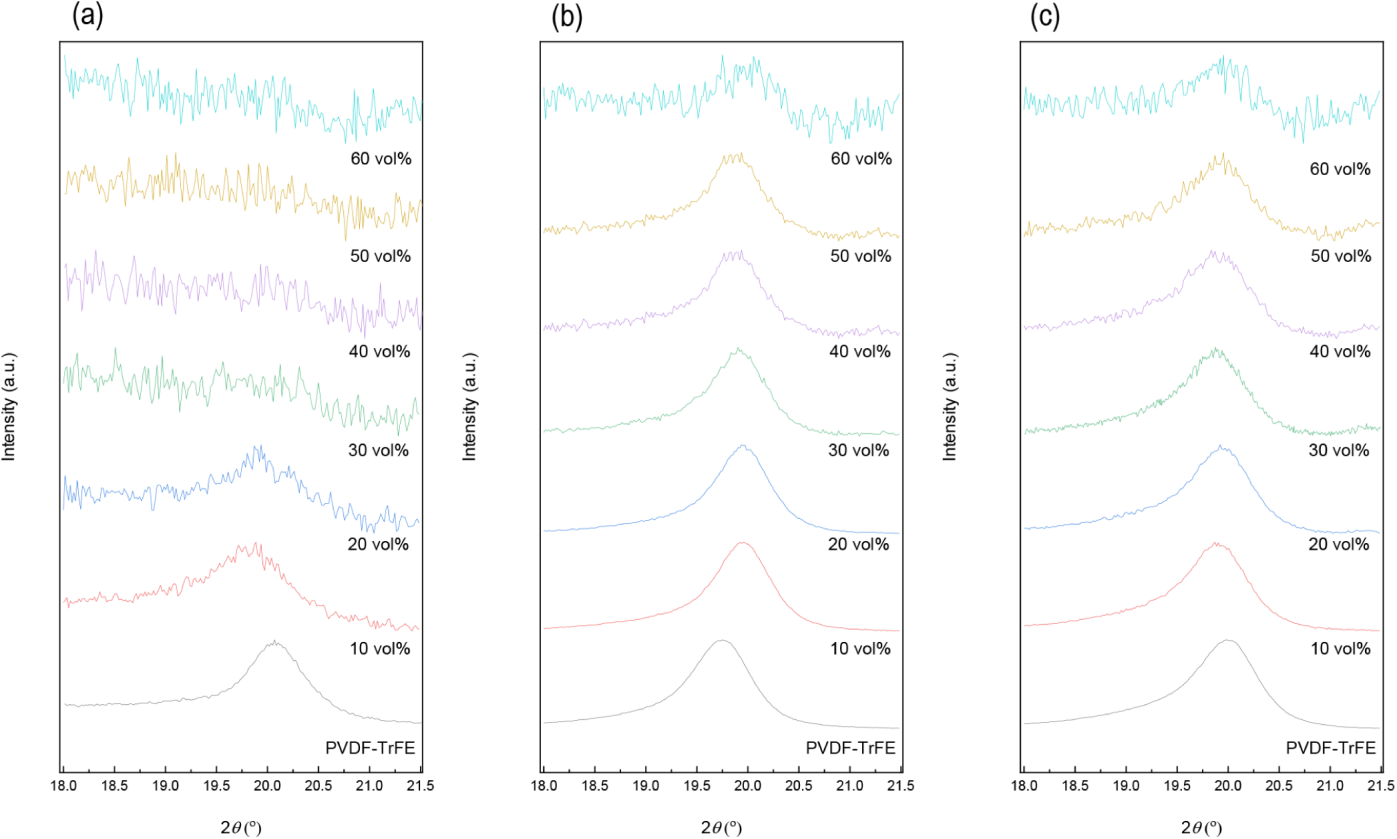
XRD patterns of (a) powder form, (b) compression molded, and (c) postprocessed samples of PVDF-TrFE and composites (10, 20, 30, 40, 50, and 60 vol %).

In the as-received powder (Figure 3a), the XRD peak is well-defined. After the dispersion of the ceramic filler, it broadens for 10 and 20 vol % reinforced composites and disappears for higher BaTiO_3_ contents. This is a consequence of amorphization, conformational disorder, and the reduction of the crystalline domain size of the polymeric matrix induced by the high forces applied during the dispersion process, which is more significant for contents above 20 vol %. After compression molding, the crystallinity is recovered. The β-phase peak can be seen in all cases, and the crystalline domain size also increases (Figure 3b). For PVDF-TrFE, the peak shifts to lower 2θ, indicative of a larger interplanar space, probably caused by the melting-crystallization process. However, the addition of BaTiO_3_ in the composite materials causes a shift of the XRD peak to higher 2θ values, indicating a smaller interplanar space. This is probably due to the strain induced in the network by the presence of ceramic particles.

The strain induced by postprocessing tends to shift the peak of the neat PVDF-TrFE to higher 2θ values (Figure 3c), which is similar to the effect of BaTiO_3_ in compression-molded composites. Another consequence of postprocessing is the reduction of the crystalline domain size (Table S1), which was also reported by Qi et al.,^31^ who observed a diminution of the crystallite size from 20 to 4.8 nm for rolled PVDF films. In contrast to the neat PVDF-TrFE, the shift caused by postprocessing is almost negligible in composite materials (Figure 3b,c). This is attributed to the presence of BaTiO_3_ particles, which, as mentioned above, constrain the movement of the polymeric chains. After postprocessing, all samples (the neat PVDF-TrFE and the composites) present a similar interplanar space (Figure 3c). Note that the macroscopic strain obtained after postprocessing (Table 1) is lower when BaTiO_3_ is incorporated, and this effect becomes significantly relevant for composite materials with 60 vol % (∼25% lower).

**Table 1:** Strain Induced by Post-Processing and Parameters Related to the Ferroelectric-Paraelectric Phase Transition, Melting, and Crystallinity

Material	Strain (%)	*T*_c1_ (°C)	*T*_m_ (°C)	Δ*H*_m_ (J/g)	*T*_cr_ (°C)	Δ*H*_cr_ (J/g)	*T*_c2_ (°C)	%*X*_*c*_
PVDF-TrFE	87.1 ± 2.2	121.1 ± 1.5	153.20 ± 0.02	28.8 ± 2.1	127.6 ± 0.5	14.7 ± 6.1	69.8 ± 0.4	27.5 ± 2.0
10 vol %	75.7 ± 0.6	117.3 ± 0.1	150.9 ± 1.0	22.2 ± 1.0	130.28 ± 0.06	15.6 ± 0.5	69.567 ±0.004	28.7 ± 1.2
20 vol %	76.3 ± 2.6	117.2 ± 0.2	149.3 ± 0.7	15.1 ± 1.1	130.0 ± 0.2	12.2 ± 0.6	68.89 ± 0.07	25.9 ± 1.8
30 vol %	78.1 ± 0.1	118.5 ± 0.4	148.1 ± 0.5	9.0 ± 0.7	128.2 ± 0.2	6.70 ± 0.05	67.9 ± 0.2	20.4 ± 1.6
40 vol %	83.0 ± 0.2	118.3 ± 0.5	149.2 ± 0.6	6.9 ± 0.6	128.9 ± 0.2	6.2 ± 0.2	68.8 ± 0.2	20.7 ± 1.8
50 vol %	78.2 ± 0.1	118.3 ± 0.6	148.1 ± 0.2	3.9 ± 0.1	128.6 ± 0.1	3.9 ± 0.2	67.5 ± 0.2	15.7 ± 0.4
60 vol %	64.9 ± 0.4	118.5 ± 1.3	146.6 ± 0.3	1.65 ± 0.05	128.1 ± 0.1	1.9 ± 0.3	64.4 ± 0.2	9.1 ± 0.3

Therefore, the only phase formed in the composite materials is the β-phase; however, increasing the content of BaTiO_3_ leads to the appearance of conformational defects and a reduction in crystallite size and crystallinity.

### 3.2 Ferroelectric-Paraelectric Phase Transition

Figure 4a shows the heating DSC curves for the studied materials after postprocessing. The ferroelectric-to-paraelectric transition on heating, which is related to the Curie temperature (*T*_c1_), occurs between 80 and 140 °C for PVDF-TrFE copolymers, depending on the TrFE molar fraction.^6,28^ In this work, 25 mol % is used, and the obtained *T*_c1_ is 121.1 ± 1.5 °C (Table 1) for the neat polymer. The addition of the ceramic phase slightly diminishes this temperature by approximately 3–4 °C and is nearly independent of the BaTiO_3_ fraction. After the ferroelectric-to-paraelectric transition, only one melting peak was observed, corresponding to the melting of the crystalline paraelectric phase, as it occurs above the *T*_c_.^32^ This temperature decreases by approximately 3–6 °C with the addition of BaTiO_3_. Although it is not strongly dependent on the BaTiO_3_ content, a reduction of approximately 4.2% was observed for high contents of 60 vol %.

**Figure 4:**
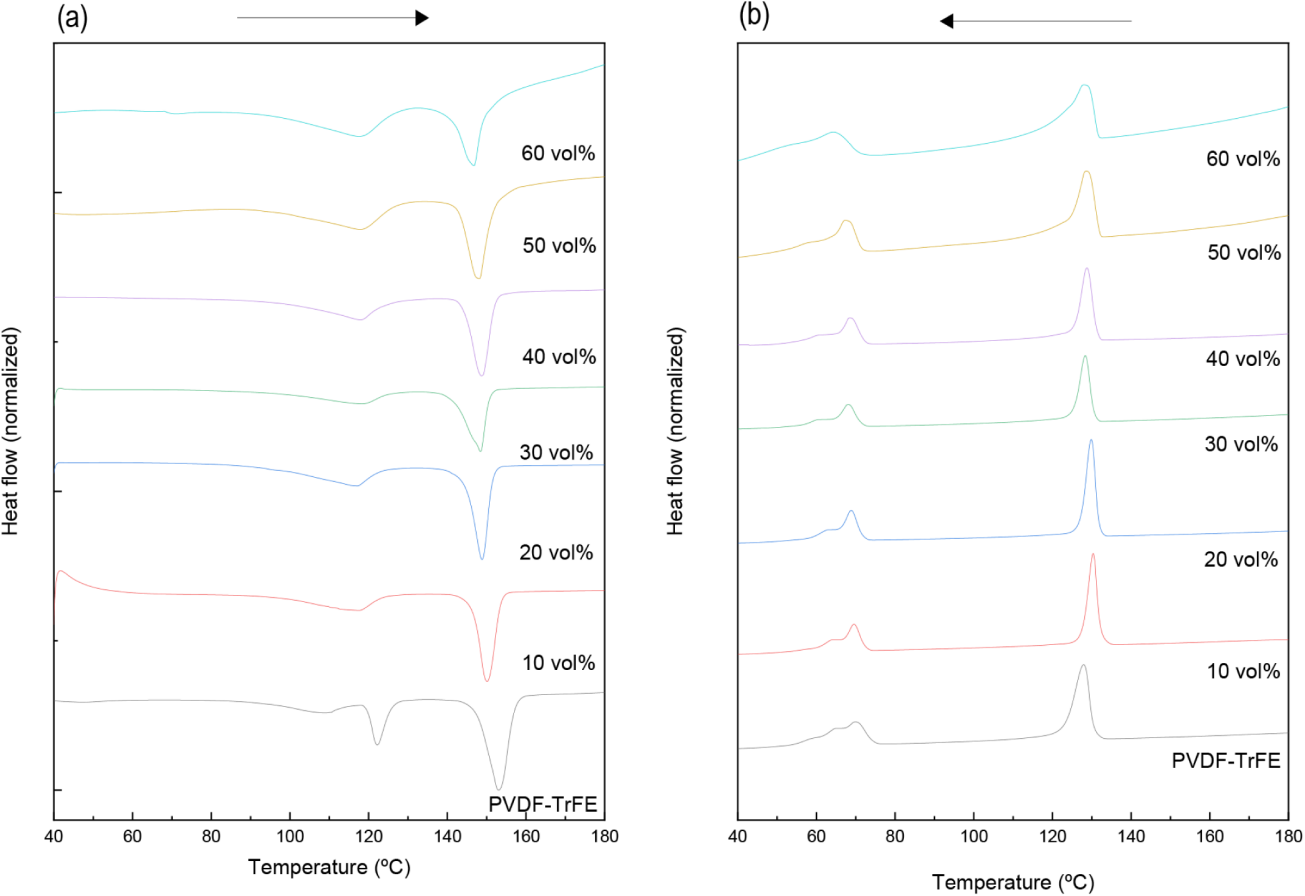
DSC curves: (a) heating and (b) cooling scans of PVDF-TrFE and composite materials after postprocessing.

The cooling DSC curves are shown in Figure 4b. Crystallization (*T*_cr_) occurs at 127.61 ± 0.45 °C for the PVDF-TrFE and increases with the incorporation of the filler (Table 1), as it acts as a nucleating agent.^33^ Additionally, the narrower peaks observed for the composite materials are related to an acceleration of the crystallization rate,^21^ which supports the assumption that ceramic particles serve as nucleation sites. The shoulder-like broadening above 50 vol % may be caused by the crystallization of different phases or conformations (β-phase, defective all-trans conformation, etc.).^30^

The Curie temperature (*T*_c2_) during the cooling process shifts to lower temperatures (∼65–70 °C) because the ferroelectric-to-paraelectric phase transition is a first-order phase transition in PVDF-TrFE.^34,35^ Additionally, the crystallinity and phase formation in PVDF-TrFE are affected by the thermal treatment and, consequently, the *T*_c_ may change after the first heating performed in DSC. It has been demonstrated that annealing above the *T*_c_ (close to 130 °C), as well as high or moderate cooling rates^25^ favor the formation of the paraelectric phase with higher crystallinity.^36^ In contrast, crystallization of the ferroelectric phases is obtained when annealing is conducted below the *T*_c_,^36^ at temperatures close to 80 °C.^25^ The different exothermic peaks observed during cooling around *T*_c2_ correspond to different phase transitions and indicate the formation of different ordered phases (different conformations), including all-trans ferroelectric and defective ferroelectric phases.^37,38^ Compared to the composite materials, these peaks are more pronounced in neat PVDF-TrFE, which means that BaTiO_3_ stabilizes one of the conformations.

The crystallization (Δ*H*_c_) and melting (Δ*H*_m_) enthalpies, as well as the crystallinity (%*X*_c_), increase slightly at a BaTiO_3_ fraction of 10 vol %, but decrease at higher contents, which is consistent with the tail observed in the XRD patterns (Figure 3c). Thus, crystallinity and preferential orientation are not promoted.^9^ Liu et al.^1^ have previously observed that %*X*_c_ decreases above a critical filler content. Abolhasani et al.^39^ also reported a diminution in crystallinity for graphene nanofiber-reinforced PVDF due to the presence of a higher number of nucleation points. The increase in BaTiO_3_ content, which increases the number of nuclei, limits the space occupied by the molecular chains of the polymer and results in spherulites with smaller dimensions.^33^ This effect becomes particularly relevant at a BaTiO_3_ fraction of 60 vol %.

### 3.3 Piezoelectric and Dielectric Properties

Regarding the piezoelectric properties, it is important to emphasize that the piezoelectric response in PVDF-TrFE and BaTiO_3_ is reversed, and under the same mechanical stress, the induced electric fields are opposite. Consequently, the *d*_*33*_ values after poling are of opposite sign: positive (*d*_*33*_ > 0) for the ceramic filler and negative (*d*_*33*_ < 0) for PVDF-TrFE, when measured in the same direction as the poling field. Therefore, quantifying the different contributions of the two components and designing effective poling procedures, while complex, is essential to maximize the overall response.

The piezoelectric coefficient of neat PVDF-TrFE as a function of the poling voltage is shown in Figure 5a. Poling was performed at room temperature (RT), and the time was always set to 5 min, as longer poling times did not result in an increase in *d*_*33*_. Measurable *d*_*33*_ values were obtained at voltages higher than 30 kV/mm and reached a value of −30.7 ± 3.5 pC/N when the poling voltage was 50 kV/mm. However, when the poling voltage was further increased to 80 kV/mm, a maximum asymptotic value of −38.7 ± 1.6 pC/N was reached (Figure S1). To isolate and estimate the piezoelectric response of BaTiO_3_ particles, a uniaxial cold-pressed sample of BaTiO_3_ was prepared using poly­(vinyl alcohol) (PVA) as a binder. PVA was used at a weight ratio of 96/4 PVA/distilled water. Note that PVA has no piezoelectric properties, and the only purpose of using it as a binder is to avoid electrical discharge during poling and to enable manipulation and measurement of the response of the ceramic powder in a well-compacted state (i.e., not sintered). The *d*_*33*_ value obtained in this case, which corresponds to the response of the ceramic skeleton composed of nanometric particles in close contact, was 29.2 ± 1.8 pC/N. In this case, the poling was performed at 4 kV/mm for 30 min at RT. In both cases, no relaxation was observed after 7 days.

**Figure 5:**
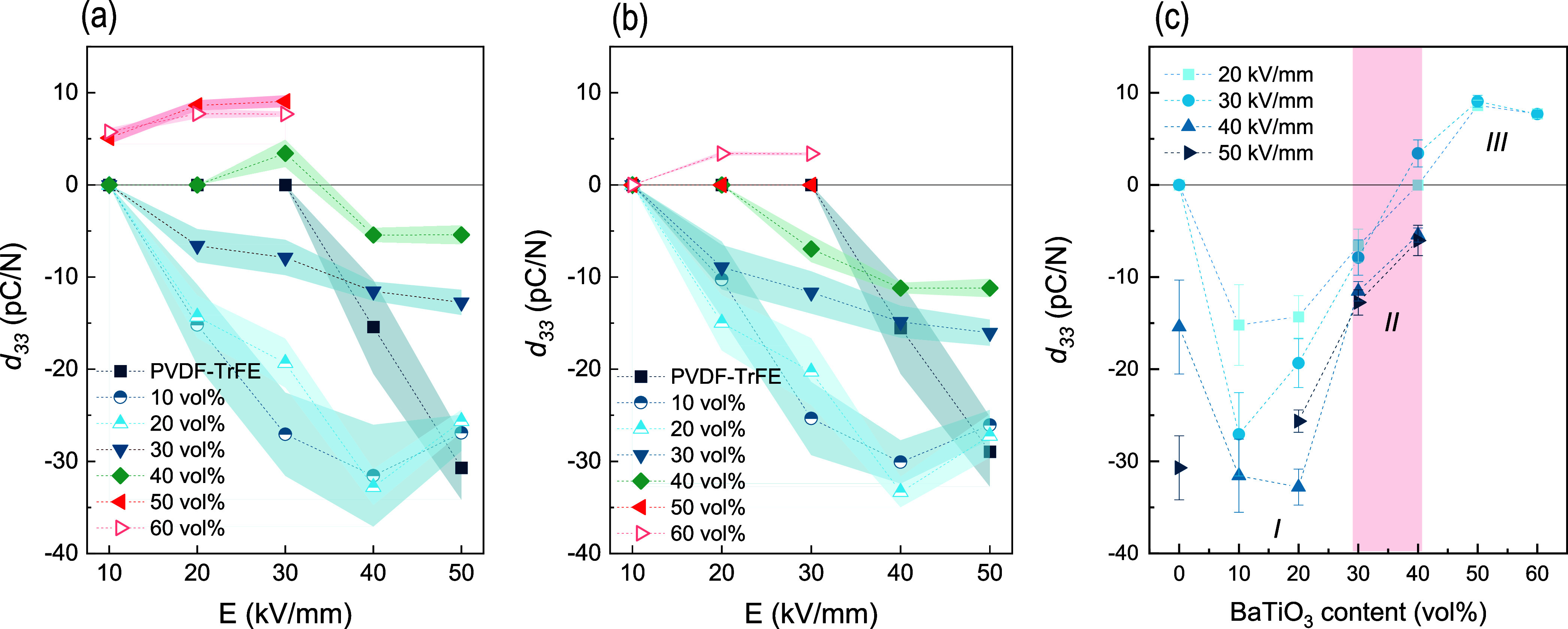
Effect of the BaTiO_3_ content and poling field on the *d*_*33*_: (a) as-poled, (b) after 24 h, and (c) percolation threshold (*II*).

The *d*_*33*_ values obtained for the different composite materials as a function of the poling voltage (*E*), applied for 5 min at RT, are also shown in Figure 5a for the as-poled samples and in Figure 5b after 24 h. A careful study of this figure allows the influence of BaTiO_3_ incorporation on the piezoelectric response of the composites to be elucidated. The presence of BaTiO_3_ significantly reduces the minimum voltage required to obtain a measurable *d*_*33*_ in all cases, but different behaviors are observed depending on the filler content. For contents below 30 vol %, a negative value of *d*_*33*_ is obtained, and poling is effective (measurable *d*_*33*_) even at 20 kV/mm. The negative response of these composites is caused by the dominant contribution of the polymer matrix, as percolation of the filler has not been reached. For example, the *d*_*33*_ values obtained for 10 and 20 vol % are very similar, approximately −15 and −35 pC/N for poling voltages of 20 and 40 kV/mm, respectively, and no relaxation was observed. More interestingly, these values of −15 and −35 pC/N are similar to those of neat PVDF-TrFE, but they are poled at 40 and 60 kV/mm, respectively. This fact implies that the incorporation of this type of filler at low contents favors the polarization of the polymer matrix. In Figure 5a, it is clearly seen that the *d*_*33*_–*E* curve for 10 and 20 vol % composites is very similar to that of PVDF-TrFE but shifted by 20 kV/mm to lower electric fields. Kim et al.^40^ have pointed out that the presence of piezoceramics well dispersed along the PVDF-TrFE matrix promotes dipole orientation at the interphases. Belovickis et al.^41^ state that ceramic phase concentrations below 20 vol % in this type of composite material do not have a relevant effect on the maximum *d*_*33*_ value because the significant difference in dielectric permittivity (*ε’*) causes the voltage not to be uniformly distributed throughout the volume. As a result, the polymer matrix is assumed to be polarized, while the ceramic particles are not, which is consistent with the results obtained in this work. Padurariu et al.^42^ have also reported that there is a locally enhanced field on the matrix (*ε’* ≈ 10), while the local field in the ceramic particles is virtually zero due to the much higher permittivity (*ε’* ≈ 10^3^). However, it is clear from Figure 5a,b that an increase in the poling voltage up to 50 kV/mm is sufficient to achieve partial polarization of the ceramic filler, as an apparent decrease in the absolute *d*_*33*_ value is obtained, caused by the opposite piezoelectric response of BaTiO_3_.

For 30 vol %-filled composites (Figure 5a), for all poling fields, there is a loss in the absolute value of *d*_*33*_ in as-poled samples, which remains negative, induced by the increasing opposite contribution of the piezoceramic response. This contribution seems to relax after 24 h, and the *d*_*33*_ value is partially recovered (Figure 5b), but it still remains lower than those for lower BaTiO_3_ contents. This can be explained not only by the contribution of the ceramic filler but also by the reduction of crystallinity in the polymer matrix (∼25%). This result suggests that the relaxation phenomenon occurs only in the piezoelectric response of BaTiO_3_ that contributes to the overall behavior of the composite material.

When the filler fraction is increased to 40 vol %, the as-poled material even exhibits a positive d_33_ at 30 kV/mm, as the piezoelectric response of the ceramic filler becomes dominant. After 24 h, relaxation of the ceramic occurs, and the *d*_*33*_ shifts to negative values. The use of higher poling fields leads to a stronger polarization of the PVDF-TrFE matrix, so that its response once again becomes the dominant contribution, and negative *d*_*33*_ values are obtained. Figures 5a,b always show lower *d*_*33*_ values for the 40 vol % composite compared to the 30 vol % composite. Since the β-phase fraction (∼100%) and crystallinity (∼20.5%) are similar in both materials, the differences are attributed to the stronger contribution of a more percolated BaTiO_3_ network. Therefore, the percolation threshold is assumed to be between 30 and 40 vol % (Figure 5c, *II*).

At higher filler concentrations (50 and 60 vol %), *d*_*33*_ switches to positive values for the entire range of poling conditions studied in this work (Figure 5a), due to the dominant contribution of the BaTiO_3_ particles and probably also to the low crystallinity (Table 1) and small crystalline domain size of the polymer matrix (Table S1). In these cases, there is also a relaxation of the positive contribution after 24 h (Figures 5b and S2). In composites with 50 vol %, a slight contribution of the PVDF-TrFE matrix is expected, as the crystallinity is relatively low (15.68 ± 0.37%), and this could be the reason for the canceled piezoelectric response after 24 h (Figure S2b). Increasing the content to 60 vol % seems to decrease the piezoelectric contribution of the polymer matrix response, probably due to the low matrix/filler ratio and the crystallinity of the matrix (<10%). Additionally, the relaxation of the positive contribution is less pronounced (Figure S2a). From these results, it seems that the low concentration of BaTiO_3_ and the presence of the PVDF-TrFE matrix favor relaxation of the piezoelectric response of the ceramic filler. Then, the relaxation diminishes with the matrix/filler ratio. This relaxation was not observed for the BaTiO_3_ compacted sample.

From the results discussed above, there are three distinct regions in terms of the piezoelectric response of the composites as a function of the BaTiO_3_ content (Figure 5c). At low BaTiO_3_ contents (Region I), *d*_*33*_ values similar to those of neat PVDF-TrFE can be obtained but with lower poling voltages of at least 20 kV/mm. The results obtained in this region are comparable to, or even higher than, those reported in the literature (Table 3). For intermediate contents (Region II), percolation seems to occur, and the contribution of the piezoelectric response of the ceramic filler becomes relevant, leading to an apparent decrease in *d*_*33*_ values, with in some cases a reversal of the sign. Higher BaTiO_3_ contents (Region III) induce the complete inversion of the response because of the dominant contribution of the piezoelectric ceramic, and the contribution of the PVDF-TrFE matrix becomes negligible for 60 vol % composites.

Typically, regardless of the ceramic content, a one-step polarization process has been used for this type of composite (Table 2), resulting in relatively low *d*_*33*_ values depending on the system and volume fractions. As an example, Lekshmipriya et al.^45^ obtained *d*_*33*_ values between 2 and 7 pC/N for 0.67BiFeO_3_–0.33BaTiO_3_ contents ranging from 10 to 60 wt % with a poling voltage of 10 kV/mm at 50 °C. However, it would be interesting to explore two-step poling strategies in materials where the piezoelectric properties of the PVDF-TrFE and the filler are observed simultaneously, as in Region II of Figure 5c. For materials included in Regions I and III, independent of the poling voltages, the observed response is dominated by the polymer matrix and the ceramic filler, respectively, and the proposed two-step poling cannot induce an increase in *d*_*33*_.

**Table 2:** Comparison of the *d*_*33*_ Values with Others Found in the Literature

Material	Filler	Content	Poling voltage/Temperature (Time)	*d*_*33*_ (pC/N)	ref.
ES-PVDF-20 (electrospinning mats)	BaTiO_3_ (<100 nm)	20 wt %	20 kV/mm—120 °C (60 min)	–18.5	^17^
PVDF-TrFE(20)-20 (foam—TIPS method)	BaTiO_3_ (0.85–1 μm)	20 wt %	20 kV/mm—120 °C (60 min)	–28.3	^28^
			–15 kV/mm—120 °C (30 min)		
PVDF-TrFE-10BT	BaTiO_3_ nanofibers	10 wt %	–15 kV/mm—120 °C (30 min)	–31.1 ± 1.3	^20^
			15 kV/mm—100 °C (10 min)		
DET-BTO/PVDF	Modified BaTiO_3_ nanofibers Di(dioctylpyrophosphato) ethylene titanate	3 wt %	40 kV/mm—RT (2 h)	40.3	^43^
			–8 kV/mm (RT) (30 min)		
BaTiO_3_/MXene/PVDF-TrFE	BaTiO_3_ (<100 nm) MXene (Ti_3_AlC_2_)	∼6 wt % 0.15 wt %	200 MV/m	15.5	^1^
BCZT/PVDF-TrFE	Aligned Ba_0.85_Ca_0.15_Ti_0.9_Zr_0.1_O_3_ nanowires	15 wt %	10 kV/mm—60 °C (8 h)	∼29	^44^
10BT/PVDF-TrFE	BaTiO_3_ (<500 nm)	10 vol % (∼26 wt %)	30 kV/mm—RT (5 min)	–25.4 ± 4.0	This work
10BT/PVDF-TrFE	BaTiO_3_ (<500 nm)	10 vol % (∼26 wt %)	40 kV/mm—RT(5 min)	–31.6 ± 5.5	This work
20BT/PVDF-TrFE	BaTiO_3_ (<500 nm)	20 vol % (∼44 wt %)	40 kV/mm—RT (5 min)	–33.3 ± 1.7	This work

In most studies, the opposite response of the piezoceramic filler is not considered or discussed, and the potential of this kind of composite material has not been fully explored. Moreover, there are other aspects that need to be considered in designing an effective poling strategy. First, the optimal poling conditions (poling voltage, time, and temperature) are different for the filler and the matrix. The poling voltages required in PVDF-TrFE are in the range of 40–100 kV/mm, while ceramics are generally polarized above *T*_c_ at 2–10 kV/mm. Additionally, when a poled piezoelectric material is heated close to *T*_c_, the relaxation becomes significant. Here, the neat polymer and composite materials have a *T*_c_ in the range of 118–121 °C. Consequently, the poling temperature cannot exceed this value, not only because it may induce a loss of *d*_*33*_ (if poled) but also because crystallinity and other structural parameters can be altered, which can have a strong impact on the piezoelectric response.

Based on these considerations, a two-step poling method was explored for the intermediate compositions (Region II), and the results are shown in Table 3. The first step focuses on the polymer matrix using optimized experimental conditions (50 kV/mm, RT, 5 min), and the second step is expected to act on the BaTiO_3_ filler and is applied with reverse polarity. Since poling at high temperatures is more efficient for the ceramic particles,^46^ the second step was performed at 100 °C, below the *T*_c_ of the PVDF-TrFE matrix (Table 1) to avoid any phase transformation and depoling, at −10 kV/mm for 30 min. The polymer is not expected to be affected by this poling voltage (Figure 5). The reverse order in the poling strategy (first the ceramic and then the polymer), although studied (Table 3), was not effective because, at 50 kV/mm (RT, 5 min), there is also polarization of the ceramic filler that partially cancels the polarization achieved in the first step.

**Table 3:** Piezoelectric Coefficient of the Different Strategies to Design Two-Step Poling

Material	Step	Poling voltage (kV/mm)	Temperature (°C)	Time (min)	*d*_*33*_ (pC/N)	*d*_*33*_ (24 h) (pC/N)
30 vol %	1	50	RT	5	–12.0 ± 1.6	–11.1 ± 0.9
	2	–10	100	30	–4,2 ± 0,9	
40 vol %	1	50	RT	5	–6.0 ± 1.7	–6.2 ± 1.5
	2	–10	100	30	–17.3 ± 0.8	
40 vol %	1	–10	100	30	7.5 ± 0.6	–5.0 ± 0.7
	2	50	RT	5	0	

For the 30 vol % composite, the enhancement is not achieved. This is probably because the BaTiO_3_ network at this content is not percolated enough, and consequently, the particles cannot be individually polarized in the second step. Additionally, it seems that the PVDF-TrFE chains are partially reoriented, favored by the relatively high temperature (100 °C) used in the process (diminution in the absolute value of *d*_*33*_). In contrast, in the 40 vol % reinforced composite, a synergistic response is achieved, increasing the value by almost 3 times in the as-poled samples after the second step. Unfortunately, this effect is lost after 24 h due to the total relaxation of the BaTiO_3_ response, and the *d*_*33*_ value from the first step is recovered. This may be due to the effect of the polarized matrix on the ceramic particles. As previously mentioned, the presence of the PVDF-TrFE matrix seems to promote relaxation of the piezoelectric response of BaTiO_3_, which was not observed in the BaTiO_3_/PVA sample.

Since one of the issues limiting the performance of PVDF-TrFE is its low permittivity, the real and imaginary parts of the complex permittivity of PVDF-TrFE and composite materials as a function of frequency at room temperature are shown in Figure 6. The incorporation of BaTiO_3_ significantly increases the permittivity over the entire frequency range, from approximately 13 to 90 (at 1 kHz), depending on the content. In all cases, there is a decrease in the real permittivity with frequency, which is more significant with increasing filler fraction and is associated with a peak in the imaginary part due to a relaxation mechanism; i.e., at higher frequencies, dipolar polarization becomes more difficult.^47^ This peak observed in the imaginary part becomes narrower, and the maximum shifts to higher frequencies as the BaTiO_3_ content increases. At the percolation region (defined as II in Figure 5), a slight deviation from the trend is observed, but the increasing tendency continues above 40 vol %.

**Figure 6:**
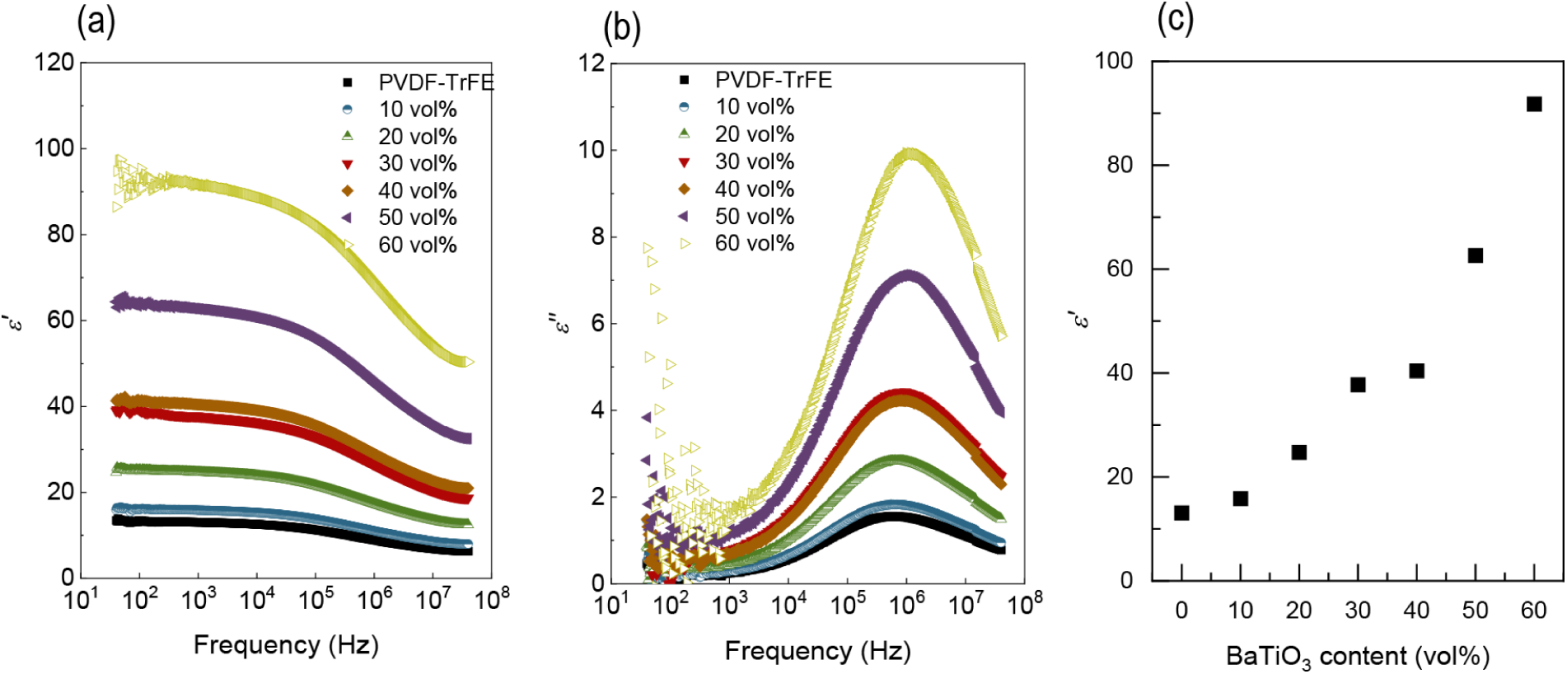
(a) Real and (b) imaginary part of the complex permittivity of PVDF-TrFE and composite materials as a function of frequency; and (c) evolution of the real part of the complex permittivity with respect to BaTiO_3_ content at 1 kHz.

## 4 Conclusions

Homogeneous dispersion of the filler was successfully achieved by high-energy ball milling for the entire range of compositions (10 to 60 vol % BaTiO_3_), avoiding the use of organic solvents, such as acetone or N,N-dimethylformamide (DMF), frequently used for this kind of materials. Obtaining the composite in powder form directly from milling is also advantageous, as it could be used as a feeding material for different processes, including additive manufacturing techniques. Additionally, all the materials studied presented β-phase fractions of ∼100%, regardless of the process and the BaTiO_3_ content.

The presence of BaTiO_3_ strongly influenced the crystallinity of the PVDF-TrFE matrix, especially for contents above 50 vol %, as the filler constrains the movement and hinders the mobility of molecular chains. The shear forces applied during milling promoted the reduction of crystallite size and induced amorphization, but crystallinity was recovered after compression molding and thermomechanical postprocessing. The presence of the ceramic particles also accelerated the crystallization as they act as nucleation sites, also increasing the temperature of the transformation.

Regarding the piezoelectric response, the addition of the ceramic filler caused a reduction in the minimum voltage required to obtain a measurable *d*_*33*_ (<20 kV/mm), which is advantageous for real applications. Three different behaviors were observed depending on the BaTiO_3_ content. For low contents (10 and 20 vol %), the contribution of the piezoelectric response of the PVDF-TrFE matrix was dominant (*d*_*33*_ < 0) due to the different dielectric permittivity of the matrix and the filler, which has been reported to induce an inhomogeneous distribution of the poling field. For intermediate contents (30 to 40 vol %), an apparent loss of the absolute value of *d*_*33*_ is induced due to the initial contribution of the opposite piezoelectric response of the ceramic filler. At high contents (50 and 60 vol %), *d*_*33*_ was dominated by the contribution of the ceramic filler and shifted to positive values. Although low BaTiO_3_ contents seem to be more attractive for maximizing *d*_*33*_, highly filled PVDF-TrFE may be interesting depending on the application due to mechanical requirements (stiffness, strength, and hardness), even if the piezoelectric response is reduced.

For intermediate compositions, where both the piezoelectric response of the matrix and the filler contribute to the overall response of the material, two-step poling was investigated. The first step focused on the polymer matrix, and the second was set to act on the BaTiO_3_. For 40 vol % reinforced composites, a synergistic effect was successfully achieved with an increase in *d*_*33*_ of approximately 180%. However, due to the effect of the polarized matrix, the piezoelectric response of the ceramic relaxed after 24 h and *d*_*33*_ for the first step was recovered. To solve this problem, interfacial engineering strategies could provide a solution to prevent relaxation phenomena.

Therefore, the present study makes it possible to conclude that the incorporation of this type of piezoceramic as a dispersed filler in this type of polymer allows the maximum value of *d*_*33*_ of the neat polymer to be obtained at significantly lower poling voltages, as well as a considerable increase in permittivity. However, the enhancement of the *d*_*33*_ value is limited due to the reverse piezoelectric response of the filler and the matrix, the relaxation of the piezoelectric ceramic, and the maximum *d*_*33*_ value reachable by the percolated network.

## Supplementary Material



## Data Availability

Data will be made available upon request.

## Notes

Structural parameters obtained from XRD of the polymer β-phase and additional data related to the piezoelectric coefficient (*d*_*33*_) (PDF)
