# Supplementary material for: Piezoelectric and Dielectric Response of BaTiO3/PVDF-TrFE Composites with High β‑Phase Content
Source: ACS Appl Polym Mater. 2025 Jun 6;7(12):7848–58. doi: 10.1021/acsapm.5c00620 (PMC12210282; doi:10.1021/acsapm.5c00620)
Supplement: Supplementary file 1 [file ap5c00620_si_001.pdf]

Supporting Information:

Piezoelectric and dielectric response of $BaTiO_3$/PVDF-TrFE composites with high β-phase content

Andrea Otero[a], María Jesús Sayagués[b], Francisco Javier Romero[a], Francisco José Gotor[b], Rocío Moriche[a,]*

[a] Departamento de Física de la Materia Condensada, Facultad de Física, Universidad de Sevilla-ICMS, Avda. Reina Mercedes, s/n, 41012, Sevilla, Spain

[b] Instituto de Ciencia de Materiales de Sevilla (ICMS), CSIC-US, Américo Vespucio, 49, 41092, Sevilla, Spain

*Corresponding author: rmoriche@us.es

Table S1. Structural parameters obtained from XRD of the polymer β-phase.

Process	Material	*2θ* (°)	*FWHM* (°)	*d* (Å)	*Crystallite size (nm)*
Powder form	PVDF-TrFE	20.072(6)	0.74(2)	4.420	12.6
	10 vol%	19.784(9)	0.91(3)	4.484	10.0
	20 vol%	19.97(8)	1.7(2)	4.442	5.0
	30 vol%	-	-	-	-
	40 vol%	-	-	-	-
	50 vol%	-	-	-	-
	60 vol%	-	-	-	-
Compression molded	PVDF-TrFE	19.751(1)	0.870(3)	4.491	10.5
	10 vol%	19.927(2)	0.699(6)	4.452	13.5
	20 vol%	19.926(3)	0.687(8)	4.452	13.8
	30 vol%	19.915(9)	0.79(3)	4.456	11.7
	40 vol%	19.91(2)	0.89(5)	4.456	10.2
	50 vol%	19.91(2)	0.89(5)	4.456	10.2
	60 vol%	-	-	-	-
Post-processed	PVDF-TrFE	19.996(1)	0.887(4)	4.437	10.3
	10 vol%	19.874(4)	0.84(4)	4.464	10.9
	20 vol%	19.944(6)	0.89(2)	4.448	10.2
	30 vol%	19.899(9)	0.98(3)	4.458	9.2
	40 vol%	19.91(2)	1.02(6)	4.455	8.8
	50 vol%	19.95(3)	1.13(9)	4.446	7.8
	60 vol%	-	-	-	-

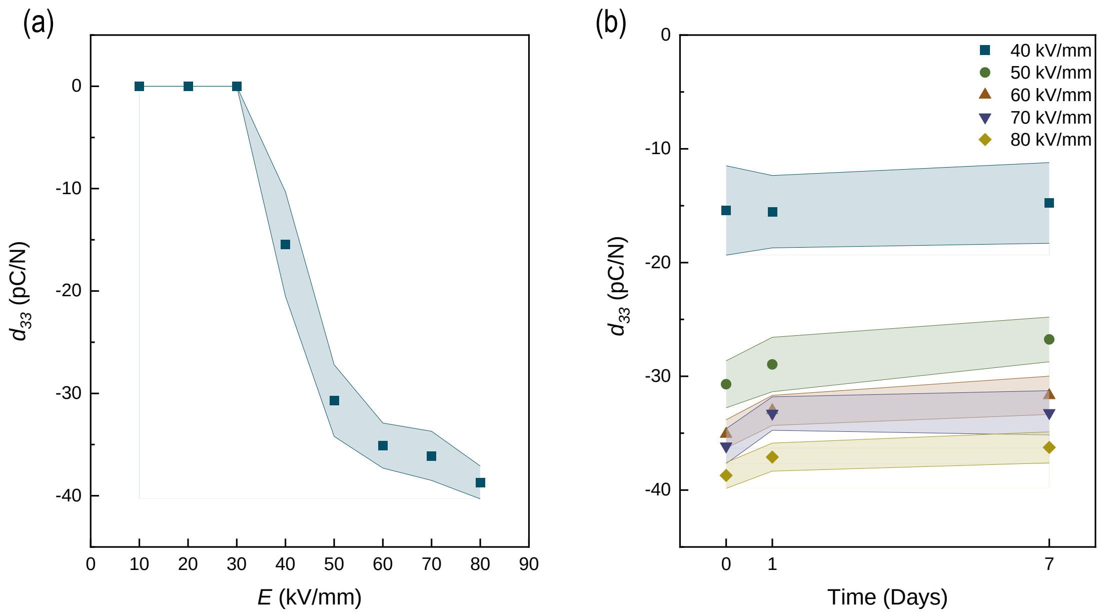

Figure S1. Piezoelectric coefficient (d_{33}) of PVDF-TrFE after post-processing: (a) effect of poling voltage and (b) relaxation depending on the poling voltage.

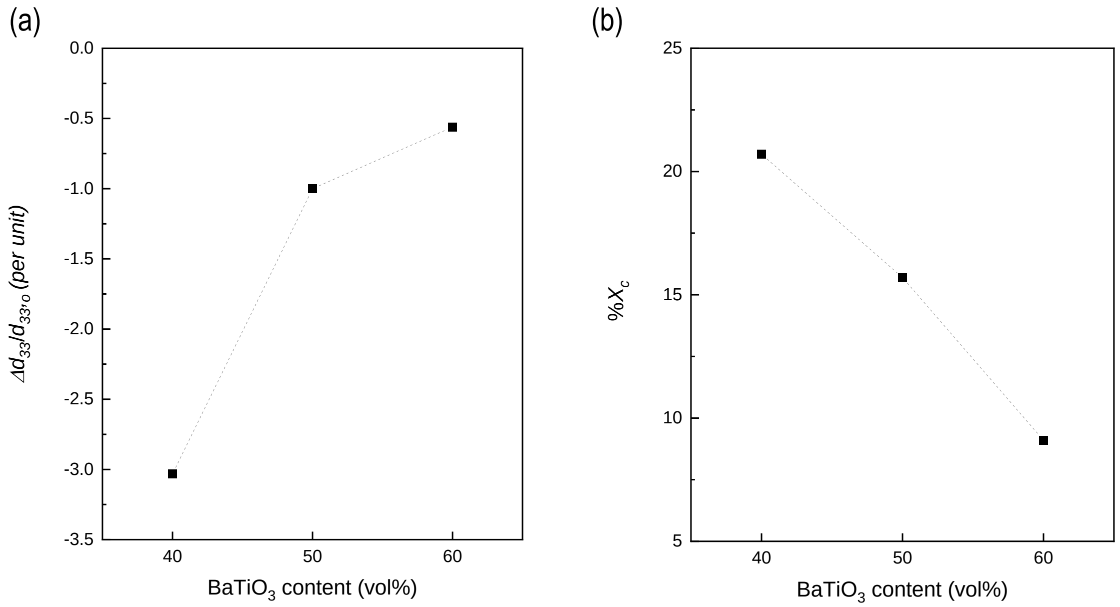

Figure S2. (a) Relaxation of the piezoelectric coefficient ($\Delta d_{33}/d_{33,0}$) after 24 h and (b) crystallinity ($\%X_c$) as a function of $BaTiO_3$ content. Contents above 40 vol% are shown as they exhibit a piezoelectric response dominated by the ceramic filler.
